# Bio-Convection Effects on Prandtl Hybrid Nanofluid Flow with Chemical Reaction and Motile Microorganism over a Stretching Sheet

**DOI:** 10.3390/nano12132174

**Published:** 2022-06-24

**Authors:** Syed Asif Ali Shah, N. Ameer Ahammad, ElSayed M. Tag El Din, Fehmi Gamaoun, Aziz Ullah Awan, Bagh Ali

**Affiliations:** 1 Department of Mathematics, University of the Punjab, Quaid-e-Azam Campus, Lahore 54590, Pakistan; asif.ali@math.uol.edu.pk; 2 Department of Mathematics and Statistics, The University of Lahore, Lahore 54000, Pakistan; 3 Department of Mathematics, Faculty of Science, University of Tabuk, P.O. Box 741, Tabuk 71491, Saudi Arabia; anaudalur@ut.edu.sa; 4 Faculty of Engineering and Technology, Future University in Egypt, New Cairo 11835, Egypt; elsayed.tageldin@fue.edu.eg; 5 Department of Mechanical Engineering, College of Engineering, King Khalid University, Abha 61421, Saudi Arabia; fgamaoun@kku.edu.sa; 6 Faculty of Computer Science and Information Technology, Superior University, Lahore 54000, Pakistan; baghalisewag@mail.nwpu.edu.cn

**Keywords:** hybrid nanofluid, bioconvection, modified Buongiorno’s model, RK-method

## Abstract

This study aims to determine the heat transfer properties of a magnetohydrodynamic Prandtl hybrid nanofluid over a stretched surface in the presence of bioconvection and chemical reaction effects. This article investigates the bio-convection, inclined magnetohydrodynamic, thermal linear radiations, and chemical reaction of hybrid nanofluid across stretching sheets. Also, the results are compared with the nanofluid flow. Moreover, the non-Newtonian fluid named Prandtl fluid is considered. Microfluidics, industry, transportation, the military, and medicine are just a few of the real-world applications of hybrid nanofluids. Due to the nonlinear and convoluted nature of the governing equations for the problem, similarity transformations are used to develop a simplified mathematical model with all differential equations being ordinary and asymmetric. The reduced mathematical model is computationally analyzed using the MATLAB software package’s boundary value problem solver, Runge-Kutta-fourth-fifth Fehlberg’s order method. When compared to previously published studies, it is observed that the acquired results exhibited a high degree of symmetry and accuracy. The velocity profiles of basic nanofluid and hybrid nanofluid are increased by increasing the Prandtl parameters’ values, which is consistent with prior observations. Additionally, the concentration and temperature of simple and hybrid nanofluids increase with the magnetic parameter values.

## 1. Introduction

The behavior of boundary layers across a stretched surface is essential because it happens in many engineering systems, such as extrusion-produced materials, paper and glass-fiber production. Polymer is constantly extruded to a windup roller from a die in industry, where it is used to make a variety of sheets and filaments. In these circumstances, the rate of cooling in the process and the stretching process determine the final product’s admirable characteristics. Researchers are currently interested in nanofluids flow across an expanding sheet.

Sakiadis [1] presented the concept of boundary layer flow over a moving solid sheet for the first time. Crane [2] is widely acknowledged as a pioneer in boundary layer flow dynamics on stretched surfaces. When a flat sheet travels linearly in its plane due to homogeneous stress, the boundary layer flow of a Newtonian fluid becomes incompressible. Gao et al. [3] investigated the analytical treatment of unsteady fluid flow between two infinite parallel surfaces of nonhomogeneous nanofluids with the help of the collocation method. Cui et al. [4] studied the influence of convection analysis of nanofluid flow over the stretched sheet with heat production and chemical reaction. Second-grade nanofluid flow through the porous sheet with activation energy, binary chemical reaction, and Marangoni limitations effects were studied by Gowda et al. [5].

The importance of heat transfer in engineering and industry has piqued the interest of researchers. In various systems, including electron devices and heat exchangers, convectional fluids such as water and ethylene glycol may be used to convey heat. However, these base liquids have low or restricted thermal conductivities. Engineers, mathematicians, and researchers from other professions are attempting to increase the thermal conductivity of the above-stated liquids by adding a single type of nanosized particle into a mixture known as ’nanofluid’, which was introduced by Choi and Eastman [6]. The ability of solid nanoparticles to boost the rate of heat transfer and thermal conductivity in convectional base fluids has been demonstrated in prior studies. As a result, many analysts and thermal experts have conducted numerical and experimental research to improve the heat transfer rate of nanofluid from various directions. For example, Tiwari and Das [7] investigated single-phase models of nanofluids. As a result, many scientists, engineers, and mathematicians have given this model strong consideration [8,9,10,11,12,13,14]. Additionally, researchers developed a novel kind of nanofluid that incorporates two different types of solid particles into a single convectional base fluid to overcome the need for better heat transfer rates in the industry and other areas. It is worth noting that in hybrid nanofluid [15], the thermal conductivity of the ordinary base fluid is higher than in basic nanofluid.

In physics, chemistry, and engineering, the study of magnetic field effects is crucial. Several metalworking procedures use the drawing of continuous filaments or strips through a quiescent fluid to cool and stretch metal strips. The techniques referred to are drawing, annealing, and thinning copper wire. Consequently, the quality of the final product is highly reliant on the rate at which these strips are dragged through an electrically conducting fluid subjected to a magnetic field and the desired feature in each of these conditions. Ali et al. [16] and Hamad [17] investigated the flow of water-based nanofluids across a stretched sheet affected by a magnetic field. Ali et al. [18] examined free convection MHD flow of viscous fluid in a vertical circular tube using damped shear and heat flux. Awan et al. [19] examined the MHD oblique stagnation point flow of second-grade fluid across an oscillating expanding sheet.

Radiation heat transfer flow is crucial for the efficient design of nuclear power plants, gas turbines, and other propulsion engines used in airplanes, missiles, satellites, and spacecraft. Consequently, Wang et al. [20] examined thermal radiation for Darcy-Forchheimer nanofluid flow using entropy. Ali et al. [21] examined the melting influence on Cattaneo-Christov and thermal radiation characteristics for aligned MHD nanofluid flows, including microorganisms across the leading edge through the FEM technique. Xiong et al. [22] investigated 2D Darcy-Forchheimer flow for hybrid nanofluids with heat sink-source and unbalanced thermal radiation effects. According to Hasona et al. [23], radiotherapy for cancer thermotherapy mainly depends on thermal radiation.

Bioconvection is a natural phenomenon that results from microorganisms’ random movement in single-cell or colony-like forms. Numerous bioconvection systems are based on the movement of microorganisms in two specific directions. For instance, when there is no movement, gyrotactic bacteria can travel in the opposite direction of gravity. Microorganisms move in a direction determined by bioconvection’s asymmetric mass distribution balance. Bioconvection is required for various bio-micro systems, including biotechnology and enzyme biosensors. A floating algae solution was introduced to demonstrate the bioconvection mechanism [24]. Plesset and Winet [25] developed the first theoretical model of bioconvection that included a diverse variety of mobile microorganisms. As a consequence of this study, Kuznetsov [26] developed a computer model to illustrate how cell deposition facilitates bioconvection growth. Waqas et al. [27] studied microorganisms in an electrically conductive viscous nanofluid on a porous stretched disc. Khan and Shehzad [28] investigated the Carreau nanofluid bioconvection flow across an expanding surface. Balla et al. [29] explored the bioconvection of oxytactic bacteria in a porous square enclosure using thermal radiation. Bioconvection is used in various fields, including pharmaceuticals, biological polymer synthesis, ecologically friendly applications, sustainable fuel cell technologies, microbial improved oil recovery, biosensors and biotechnology, and mathematical modeling enhancements.

We observed no study on bio-convective Prandtl hybrid nanofluid flow in the literature. The aim of the current article is to boost the heat transfer rate. The novelties of our research are: (i) Prandtl non-Newtonian fluid is considered, (ii) how effect inclined MHD, Brownian motion and thermophoresis diffusion, and motile microorganism on fluid flow, (iii) convective boundary effect is also considered, and (iv) nanofluid and hybrid nanofluid flow results are compared.

In this investigation, the following scientific research questions are answered:

1. What is the impact of multi-buoyancy forces, inclined magnetic field, and Prandtl parameters on the fluid velocity subject to mono and hybrid nanofluids flow?
2. What is the effect of the magnetic field, Prandtl parameters, Brownian motion, and thermophoresis on the temperature and heat transfer rate for mono and hybrid nanofluid flow?
3. How is the concentration affected by the magnetic field, Lewis number, chemical reaction parameter, Brownian motion, and thermophoresis for mono and hybrid nanofluid flow?
4. Determine how bio-convection influences motile dispersion and mass transfer of motile microbe density?

## 2. Mathematical Formulation

**Figure 1:**
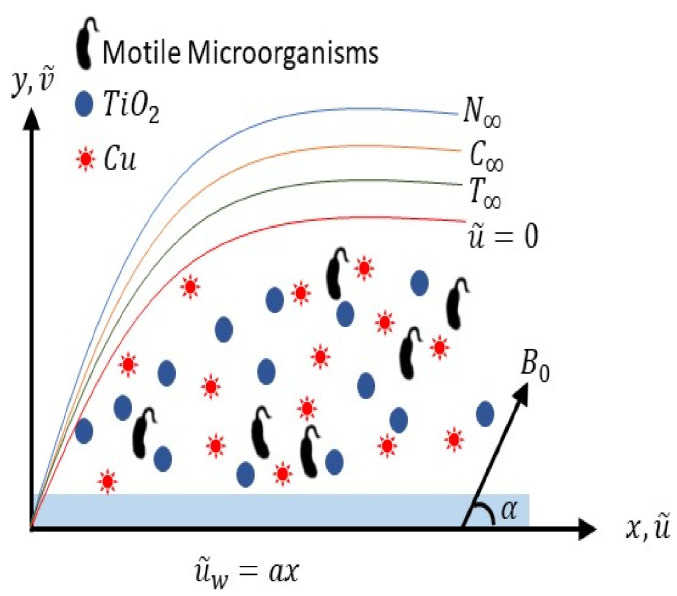
Problem’s geometry.

Considered Prandtl hybrid nanofluid with two-dimensional incompressible steady flow due to an expanding sheet with motile microorganisms. The coordinate system (x,y) is chosen that is perpendicular and flow is assumed at y>0. An inclined magnetic field is applied to the fluid flow, which makes an angle α with the *x*-axis and ${\hat{u}}_{w}\left(x\right)=ax$ (*a* is constant) is the velocity with which the plate is expanded along the *x*-axis as shown in Figure 1.

The governing equations of Prandtl hybrid nanofluid are given as ([30,31]):

(1) $$\mathrm{Continuity}\;\mathrm{Equation}:\frac{\partial \tilde{v}}{\partial y}+\frac{\partial \tilde{u}}{\partial x}=0,$$

$$\mathrm{Momentum}\;\mathrm{Equation}:\;\tilde{v}\frac{\partial \tilde{u}}{\partial y}+\tilde{u}\frac{\partial \tilde{u}}{\partial x}=\nu_{hnf}\frac{A}{C}\frac{\partial^{2}\tilde{u}}{\partial y^{2}}+\nu_{hnf}\frac{A}{2C^{3}}{\left(\frac{\partial \tilde{u}}{\partial y}\right)}^{2}\frac{\partial^{2}\tilde{u}}{\partial y^{2}}-\frac{\sigma_{hnf}B_{0}^{2}\tilde{u}}{\rho_{hnf}}\sin^{2}\left(\alpha \right)$$

(2) $$+\frac{1}{\rho_{hnf}}\left[\overset{´}{g}\left(T-T_{\infty }\right)\rho_{f}\overset{´}{\beta }\left(1-C_{\infty }\right)+\overset{´}{g}\left(C_{\infty }-C\right)\left(\rho_{p}+\rho_{f}\right)-\left(\rho_{m}-\rho_{p}\right)\left(N_{\infty }-N\right)\overset{´}{g}\gamma^{*}\right],$$

(3) $$\mathrm{Energy}\;\mathrm{Equation}:\;\tilde{v}\frac{\partial T}{\partial y}+\tilde{u}\frac{\partial T}{\partial x}={\bar{\alpha }}_{hnf}\frac{\partial^{2}T}{\partial y^{2}}+\tau \left[{\left(\frac{\partial T}{\partial y}\right)}^{2}\frac{D_{T}}{T_{\infty }}+\frac{\partial T}{\partial y}D_{B}\frac{\partial C}{\partial y}\right],$$

(4) $$\mathrm{Concentration}\;\mathrm{Equation}:\;\tilde{v}\frac{\partial C}{\partial y}+\tilde{u}\frac{\partial C}{\partial x}=\frac{\partial^{2}C}{\partial y^{2}}D_{B}+\left(\frac{\partial^{2}T}{\partial y^{2}}\right)\frac{D_{T}}{T_{\infty }}+K_{r}\left(C_{\infty }-C\right),$$

(5) $$\mathrm{Motile}\;\mathrm{Microorganism}\;\mathrm{Equation}:\;\;\;\;\;\;\;\tilde{v}\frac{\partial N}{\partial y}+\tilde{u}\frac{\partial N}{\partial x}=D_{m}\frac{\partial^{2}N}{\partial y^{2}}+\frac{bw_{c}}{\left(C_{\infty }-C_{w}\right)}\frac{\partial }{\partial y}\left(N\frac{\partial C}{\partial y}\right).$$

All the involved terms in these equations are defined in nomenclature. The following are the suitable boundary limits [32]:

(6) $$\begin{array}{c} \mathrm{when}\;y=0,\tilde{v}=0,{\tilde{u}}_{w}=\tilde{u}=ax,\frac{\partial T}{\partial y}=-\left(T_{w}-T\right)\frac{h}{k_{hnf}},C_{w}=C,N_{w}=N, \end{array}$$

(7) $$\begin{array}{c} \mathrm{when}\;y\to \infty \;C_{\infty }=C,\tilde{u}=0,N_{\infty }=N,T_{\infty }=T. \end{array}$$

By utilizing the similarity relations given below, the above PDEs can be transformed into ODEs [33]

(8) $$\eta =\sqrt{\frac{a}{\nu_{f}}}y,\theta \left(\eta \right)=\frac{T-T_{\infty }}{T_{w}-T_{\infty }},\phi \left(\eta \right)=\frac{C_{\infty }-C}{C_{\infty }-C_{w}},\chi \left(\eta \right)=\frac{N-N_{\infty }}{N_{w}-N_{\infty }},\psi^{†}=f\left(\eta \right)\sqrt{a\nu_{f}}x,$$

where

$$\tilde{u}=\frac{\partial \psi^{†}}{\partial y},\;\;\;\tilde{v}=-\frac{\partial \psi^{†}}{\partial x}.$$

By the above similarity relations Equation (1) is justified identically and Equations (2)–(5) are rewritten as:

(9) $$f^{\prime \prime \prime }\left(\alpha_{1}+\alpha_{2}f^{\prime \prime }\right)-B_{2}f^{\prime }M\sin^{2}\alpha +B_{1}\left(ff^{\prime \prime }-f^{\prime 2}\right)+B_{2}\left(\theta -Nr\phi -Nc\chi \right)\lambda =0,$$

(10) $$\frac{1}{PrB_{3}B_{4}}\theta^{\prime \prime }+\theta^{\prime }\phi^{\prime }Nb+f\theta^{\prime }+\theta^{\prime 2}Nt=0,$$

(11) $$\phi^{\prime \prime }+fLe\phi^{\prime }+\frac{Nt}{Nb}\theta^{\prime \prime }-\gamma \phi =0,$$

(12) $$\chi^{\prime \prime }+fLb\chi^{\prime }-\left(\chi^{\prime }\phi^{\prime }+\left(\varpi +\chi \right)\phi^{\prime \prime }\right)Pe=0,$$

and Equations (6) and (7) become

(13) $$\mathrm{when}\;\eta =0;f^{\prime }=1,f=0,\chi =1,\theta^{\prime }=\frac{k_{f}}{k_{hnf}}Bi\left(\theta -1\right),\phi =1.$$

(14) $$\mathrm{when}\;\eta \to \infty ;\phi \to 0,\theta \to 0,\chi \to 0,f^{\prime }\to 0,$$

where ϕ, *f* , θ, and χ are functions of η. Moreover, prime stands for differentiation, $M=\frac{\sigma_{f}B_{0}^{2}}{\rho_{f}a}$ denotes the magnetic parameter, $\alpha_{1}=\frac{A}{C}$ is Prandtl fluid parameter, $\alpha_{2}=\frac{a^{3}x^{2}A}{2\nu_{f}C^{3}}$ denotes elastic parameter, the buoyancy ratio parameter is expressed as $Nr=\frac{\left(\rho_{p}-\rho_{f}\right)\left(C_{w}-C_{\infty }\right)}{\rho_{f}\overset{´}{\beta }\left(1-C_{\infty }\right)\left(T_{w}-T_{\infty }\right)}$, $Nt=\frac{\left(T_{w}-T_{\infty }\right)\tau D_{T}}{\nu_{f}T_{\infty }}$ is the parameter of thermophoresis, $Bi=\frac{h}{k_{f}}\sqrt{\frac{\nu_{f}}{a}}$ is thermal Biot number, $Nb=\frac{\tau D_{B}\left(C_{w}-C_{\infty }\right)}{\nu_{f}}$ shows the Brownian motion parameter, $\lambda =\frac{\left(1-C_{\infty }\right)\overset{´}{\beta }\overset{´}{g}\left(T_{w}-T_{\infty }\right)}{ax^{2}}$ indicates mixed convection parameter, $\frac{\nu_{f}}{D_{B}}=Le$ signifies the Lewis number, $Nc=\frac{\gamma^{*}\left(\rho_{m}-\rho_{f}\right)\left(N_{\infty }-N_{w}\right)}{\left(C_{\infty }-1\right)\rho_{f}\overset{´}{\beta }\left(T_{w}-T_{\infty }\right)}$ indicates Rayleigh number of bioconvection, $\gamma =\frac{k_{0}}{a}Le$ is a chemical reaction parameter, $Lb=\frac{\nu_{f}}{D_{m}}$ denotes bioconvection Lewis number, $\varpi =\frac{N_{\infty }}{N_{w}-N_{\infty }}$ indicates bioconvection constant, $Pr=\frac{\nu_{f}{\left(\rho C_{p}\right)}_{f}}{k_{f}}$ shows the Prandtl number, and $Pe=\frac{bw_{c}}{D_{m}}$ be Peclet number.

The thermo-physical attributes are given in Table 1 and Table 2.

Also

(15) $$B_{1}=\left[\left(\left(1-\phi_{1}\right)+\phi_{1}\frac{\rho_{s1}}{\rho_{f}}\right)\left(1-\phi_{2}\right)+\phi_{2}\frac{\rho_{s2}}{\rho_{f}}\right]{\left(1-\phi_{2}\right)}^{2.5}{\left(1-\phi_{1}\right)}^{2.5},$$

(16) $$B_{2}={\left(1-\phi_{2}\right)}^{2.5}{\left(1-\phi_{1}\right)}^{2.5},$$

(17) $$B_{3}=\left(\left(1-\phi_{1}\right)+\phi_{1}\frac{{\left(\rho c_{p}\right)}_{s1}}{{\left(\rho c_{p}\right)}_{f}}\right)\left(1-\phi_{2}\right)+\frac{{\left(\rho c_{p}\right)}_{s2}}{{\left(\rho c_{p}\right)}_{f}}\phi_{2},$$

(18) $$B_{4}=\frac{\left(k_{bf}-k_{s2}\right)\phi_{2}+k_{s2}+\left(m-1\right)k_{bf}}{\left(m-1\right)k_{bf}+k_{s2}-\left(m-1\right)\phi_{2}\left(k_{bf}-k_{s2}\right)}.\frac{k_{s1}+\left(m-1\right)k_{f}+\phi_{1}\left(k_{f}-k_{s1}\right)}{-\left(m-1\right)\left(k_{f}-k_{s1}\right)\phi_{1}+k_{s1}+\left(m-1\right)k_{f}}.$$

The following are the definitions for the physical quantities [32]:

(19) $${\tilde{C}}_{fx}=\frac{\tau_{w}}{\rho_{f}{\tilde{u}}_{w}^{2}},\tilde{N}u_{x}=\frac{xq_{w}^{*}}{k_{f}\left(T_{w}-T_{\infty }\right)}.$$

At the surface $\tau_{w}=\mu_{hnf}\left(\frac{A}{C}\frac{\partial \tilde{u}}{\partial y}+\frac{A}{2C^{3}}{\left(\frac{\partial \tilde{u}}{\partial y}\right)}^{3}\right)$ is shear stress and $q_{w}^{*}=-k_{hnf}{\left(\frac{\partial T}{\partial y}\right)}_{y=0}$ is heat flux.

Utilizing the predefined similarity relations, expressions (19) become

(20) $${\tilde{C}}_{fx}=\frac{1}{B_{2}}Re_{x}^{-0.5}\left(\alpha_{1}+\alpha_{2}f^{\prime \prime }{\left(0\right)}^{2}\right)f^{\prime \prime }\left(0\right),$$

(21) $$\tilde{N}u_{x}=-\frac{k_{hnf}}{k_{f}}Re_{x}^{0.5}\theta^{\prime }\left(0\right),$$

here, $Re_{x}=\frac{{\tilde{u}}_{w}x}{\nu_{f}}$ pertains Reynolds number.

## 3. Numerical Solution

Due of the nonlinear nature of the DEs (9–12), with the limits (13, 14), these are unable to be solved analytically. We use Runge-Kutta approach to solve the given problem numerically. The problem presented here illustrates ODEs with boundary conditions. At the start of the process, the coupled nonlinear ODEs (9–12) are simplified into first order DEs utilizing the following procedure:

$$f^{\prime }=z_{2},\;f=z_{1},\;f^{\prime \prime }=z_{3},\;f^{\prime \prime \prime }=zz_{1},\;\theta =z_{4},\;\theta^{\prime }=z_{5},\;\theta^{\prime \prime }=zz_{2},\;\phi =z_{6},\;\phi^{\prime }=z_{7},\;\phi^{\prime \prime }=zz_{3},\;\chi =z_{8},\;\chi^{\prime }=z_{9},\;\chi^{\prime \prime }=zz_{4}$$

(22) $$zz_{1}=\frac{1}{\left(\alpha_{1}+\alpha_{2}z_{3}\right)}\left(B_{2}z_{2}M\sin^{2}\alpha -B_{1}\left(z_{1}z_{3}-z_{2}^{2}\right)-B_{2}\lambda \left(z_{4}-Nrz_{5}-Ncz_{8}\right)\right),$$

(23) $$zz_{2}=-PrB_{3}B_{4}\left(Nbz_{5}z_{7}+z_{1}z_{5}+Ntz_{5}^{2}\right),$$

(24) $$zz_{3}=-Lez_{1}z_{7}-\frac{Nt}{Nb}zz_{2}+\gamma z_{6},$$

(25) $$zz_{4}=-Lbz_{1}z_{9}+Pe\left(zz_{3}\left(z_{8}+\varpi \right)+z_{7}z_{9}\right),$$

along with the boundary conditions:

(26) $$z_{2}=0,z_{1}=0,z_{5}=\frac{k_{f}}{k_{hnf}}Bi\left(z_{4}-1\right),z_{6}=1,z_{8}=1\;\mathrm{at}\;\eta =0,$$

(27) $$z_{8}\to 0,z_{6}\to 0,z_{4}\to 0,z_{2}\to 0\;\mathrm{at}\;\eta \to \infty .$$

The Runge-Kutta (RK) method is used to predict omitted initial conditions. This method helps in determining the missing initial conditions, such that conditions at η→∞ are satisfied. Finally, the required numerical solutions are found utilizing the fourth-order RK technique given below:

(28) $$s_{k+1}=s_{k}+h\left(\frac{25}{216}k_{1}+\frac{1408}{2565}k_{2}+\frac{2197}{4104}k_{3}-\frac{1}{5}k_{4}\right),$$

where

(29) $$k_{0}=g\left(t_{k},s_{k}\right),$$

(30) $$k_{1}=g\left(t_{k}+\frac{h}{4},s_{k}+\frac{hk_{0}}{4}\right),$$

(31) $$k_{2}=g\left(t_{k}+\frac{3h}{8},s_{k}+h\left(\frac{3k_{0}}{32}+\frac{9k_{1}}{32}\right)\right),$$

(32) $$k_{3}=g\left(t_{k}+\frac{12h}{13},s_{k}+h\left(\frac{1932k_{0}}{2197}-\frac{7200k_{1}}{2197}+\frac{7296k_{2}}{2197}\right)\right),$$

(33) $$k_{4}=g\left(t_{k}+h,s_{k}+h\left(\frac{439k_{0}}{216}-8k_{1}+\frac{3860k_{2}}{513}+\frac{845k_{3}}{4104}\right)\right).$$

A solution for Prandtl hybrid nanofluid over an expanding surface is produced by running the mathematical coding described earlier via a Matlab script. The numerical findings of physical parameters such as the Nusselt number $-\theta^{{}^{\prime }}\left(0\right)$ and the skin friction factor $-f^{\prime \prime }\left(0\right)$ are analyzed here. The computational results of the suggested technique (RK-5) are compared with those obtained by various researchers [34,35,36,37,38,39,40] under limiting circumstances. Table 3 and Table 4 reveal that the current results are compatible.

## 4. Results and Discussion

**Figure 2:**
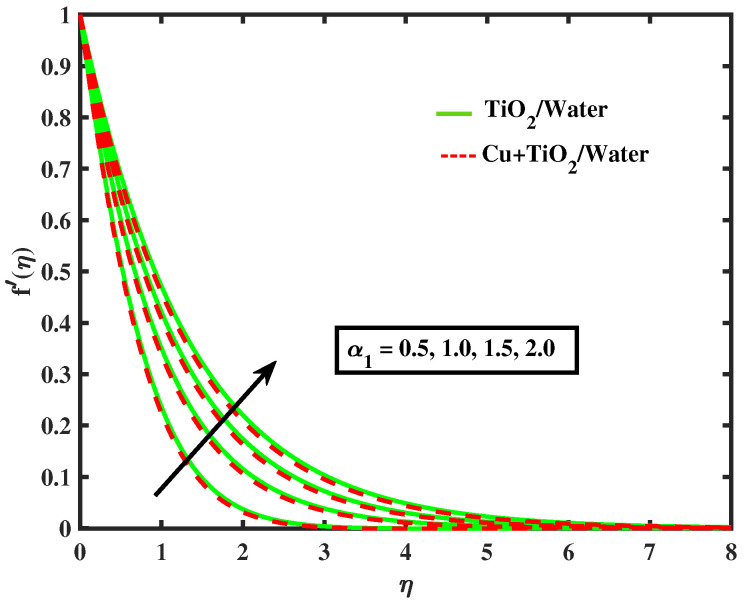
Variations of $f^{\prime }$ versus $\alpha_{1}$.

**Figure 3:**
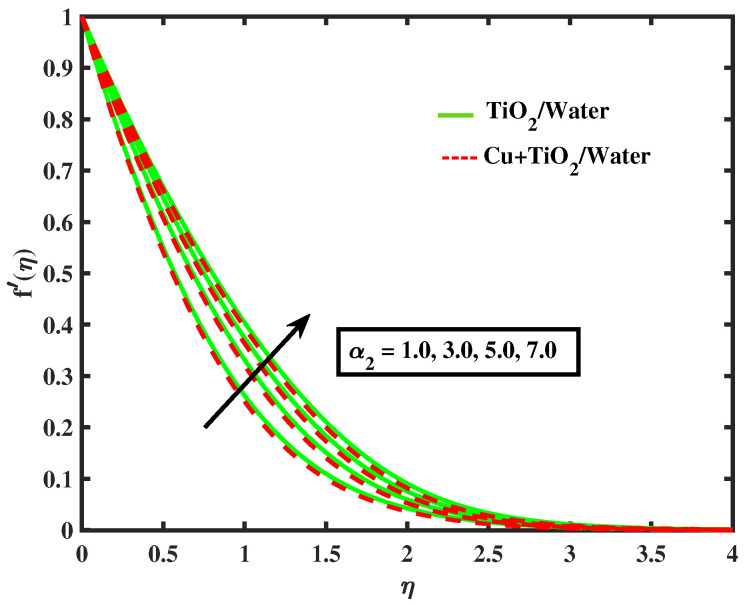
Variations of $f^{\prime }$ versus $\alpha_{2}$.

**Figure 4:**
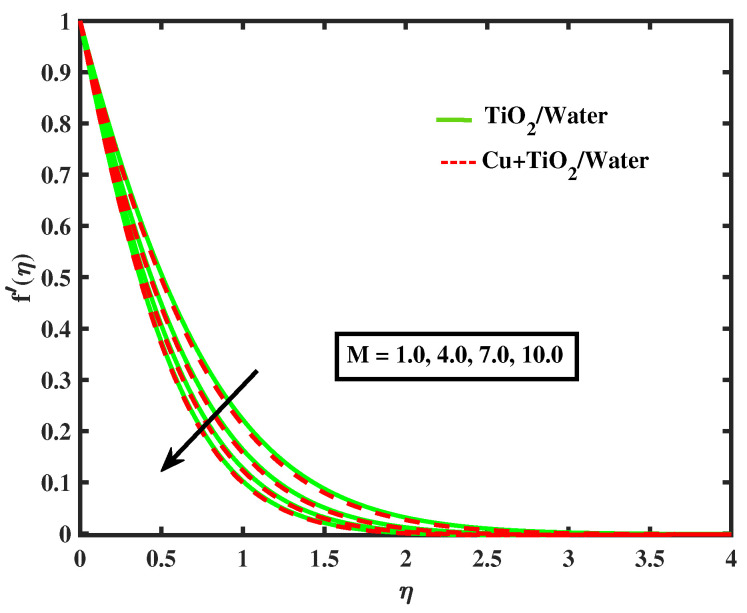
Variations of $f^{\prime }$ versus *M*.

**Figure 5:**
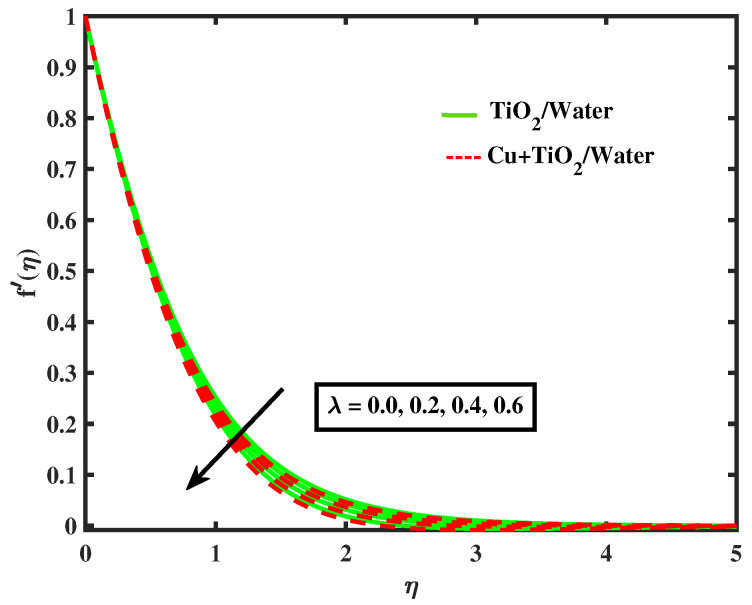
Variations of $f^{\prime }$ versus λ.

**Figure 6:**
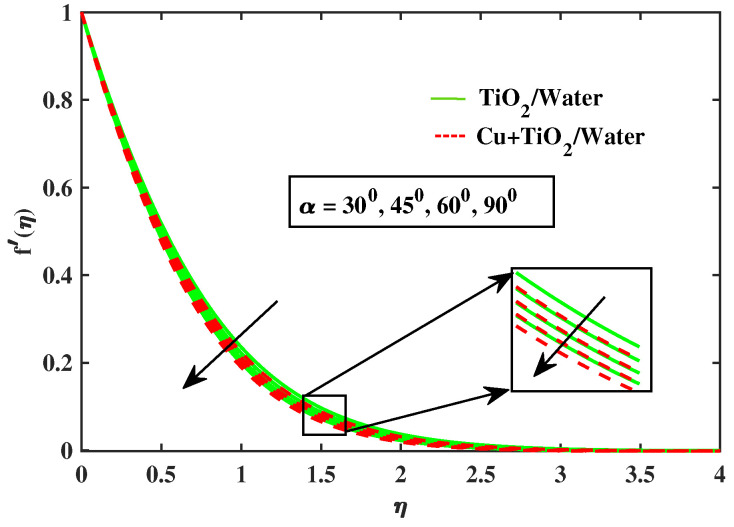
Variations of $f^{\prime }$ versus α.

**Figure 7:**
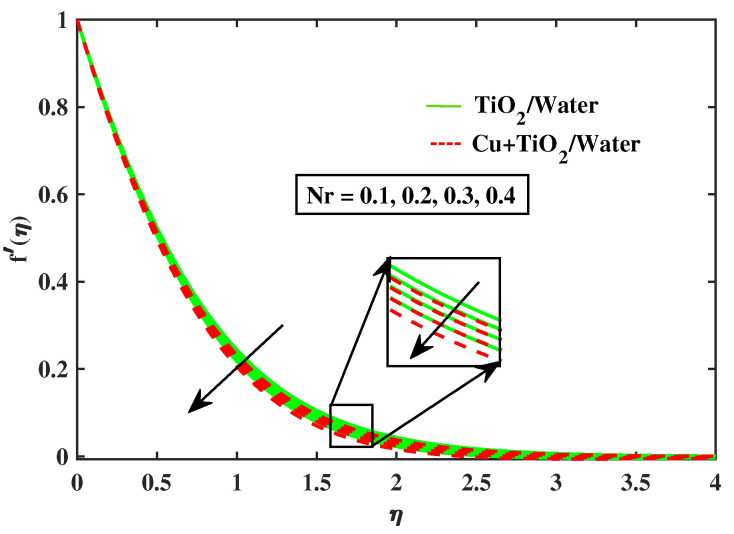
Variations of $f^{\prime }$ versus Nr.

**Figure 8:**
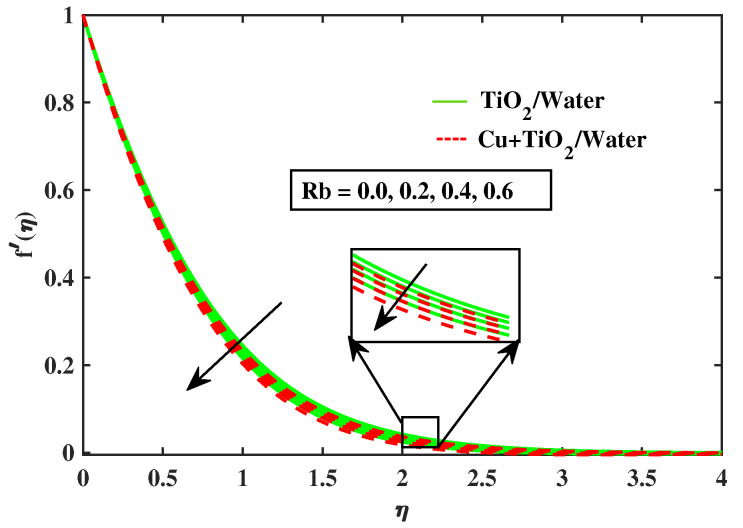
Variations of $f^{\prime }$ versus Rb.

Numerical results of physical parameters for two cases of fluid flow are determined as follows: a = $TiO_{2}$/Water (simple nanofluid) and b = $Cu+TiO_{2}$/Water (hybrid nanofluid). The above results are verified when compared with previous Pr results in limiting cases, as shown in Table 4. Each physical parameter such as velocity, temperature, concentration, and microorganism are evaluated numerically by giving predetermined values to all of the other factors involved. All figs. presented results of two type flows such as single nanofluid ($TiO_{2}$/Water) flow and hybrid nanofluid ($Cu+TiO_{2}$/Water) flow. Figure 2 and Figure 3 illustrate the impact of Prandtl fluid parameter ($\alpha_{1}$) and elastic parameter ($\alpha_{2}$) on velocity profile. The velocity profile of simple nanofluid and hybrid nanofluid increase by increasing the value of ($\alpha_{1}$) and ($\alpha_{2}$). This occurs because boosting the Prandtl fluid parameter reduces fluid viscosity. As a result of higher Prandtl fluid values, fluid becomes less viscous, and velocity profiles increase. Figure 4, Figure 5, Figure 6, Figure 7 and Figure 8 show the effects of M,λ,α,Nr and Rb on the velocity of simple nanofluid and hybrid nanofluid flow. The velocity profiles for simple and hybrid nanofluid flow decline by boosting the values of all parameters. By boosting *M*, the Lorentz forces slowdown the fluid motion.

**Figure 9:**
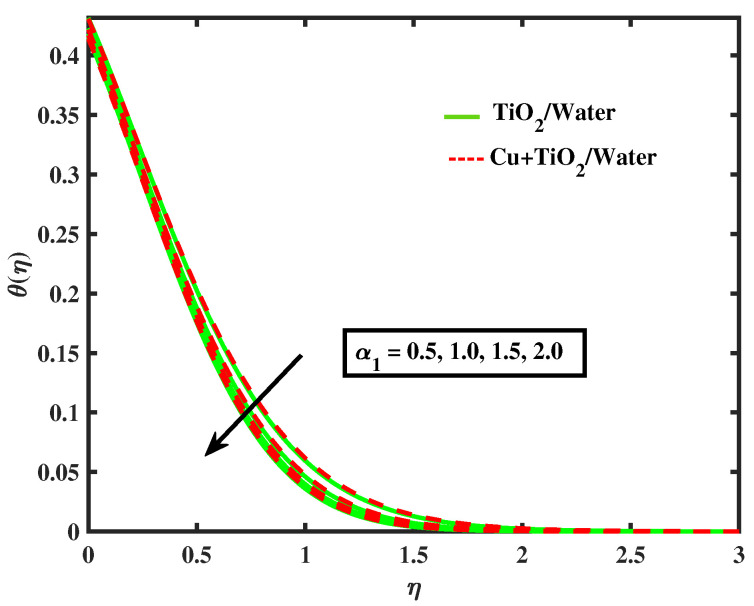
Variations of θ versus $\alpha_{1}$.

**Figure 10:**
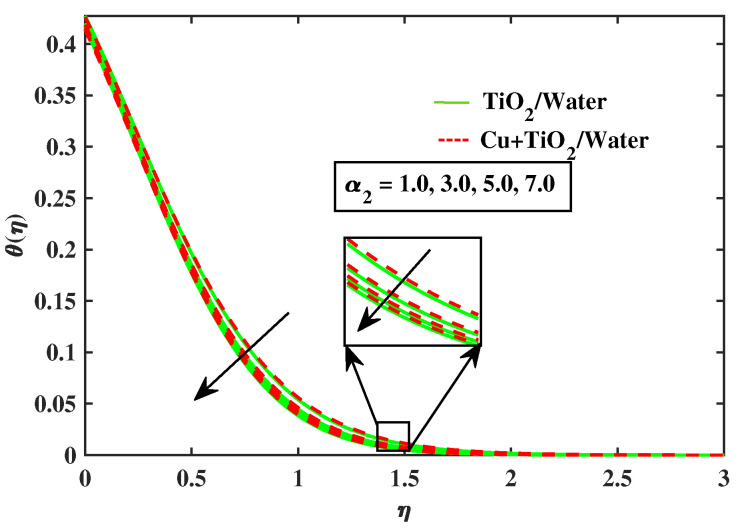
Variations of θ versus $\alpha_{2}$.

**Figure 11:**
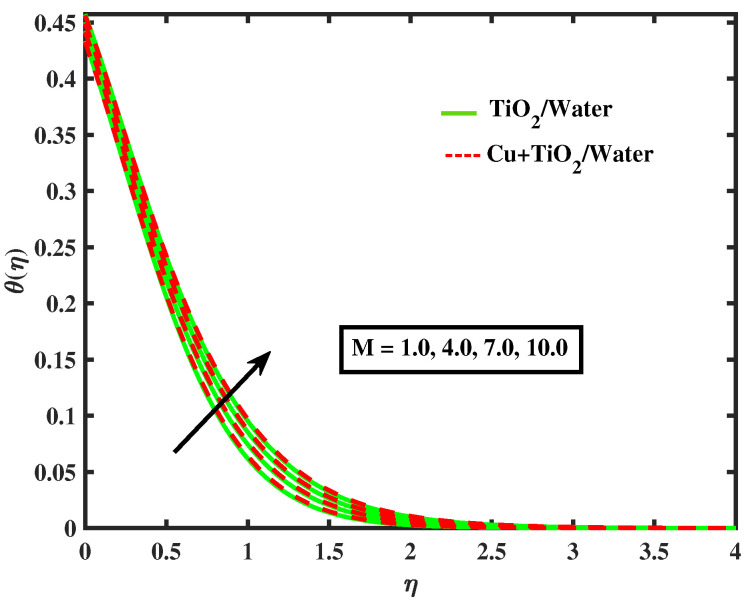
Variations of θ versus *M*.

**Figure 12:**
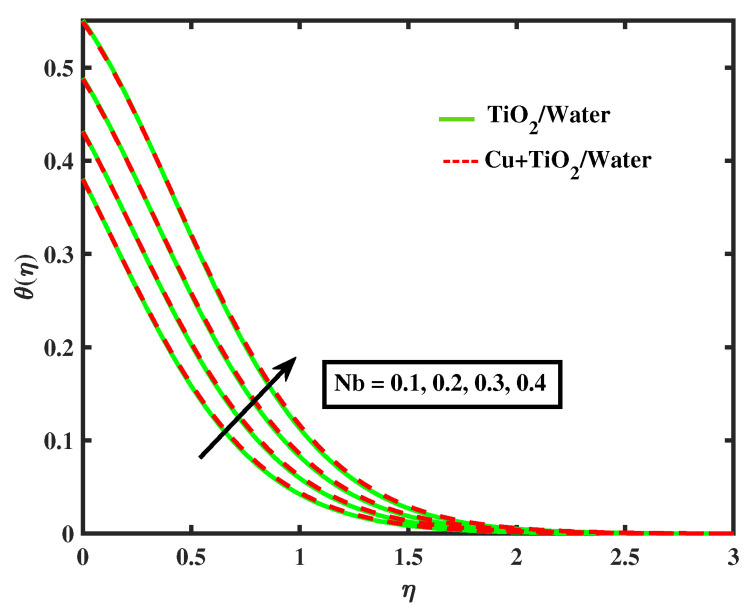
Variations of θ versus Nb.

**Figure 13:**
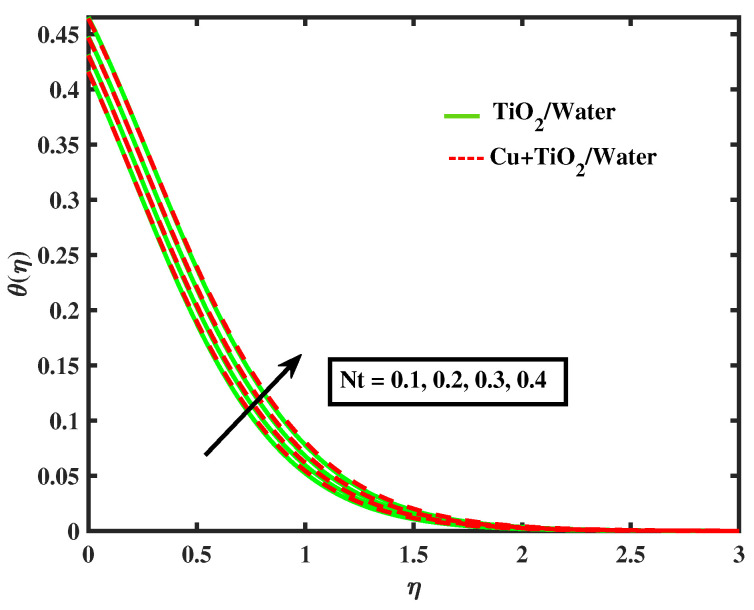
Variations of θ versus Nt.

**Figure 14:**
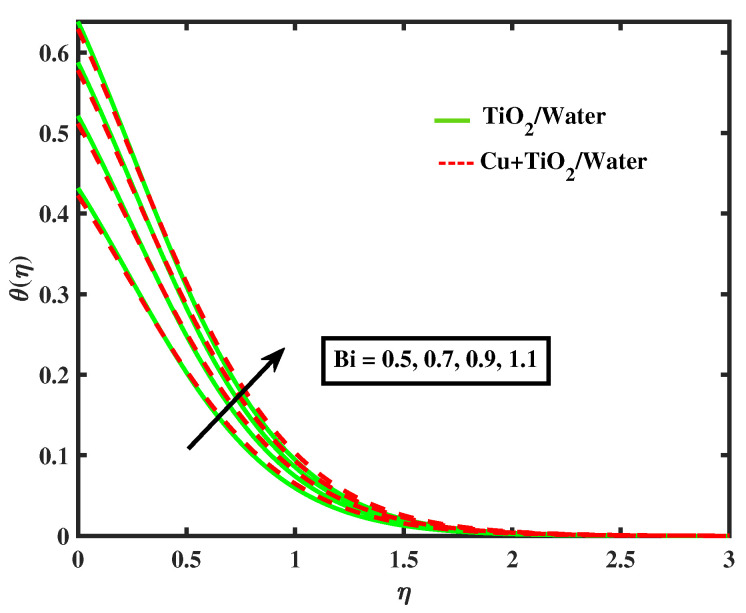
Variations of θ versus Bi.

The impacts of ($\alpha_{1}$) and ($\alpha_{2}$) on temperature is illustrated in Figure 9 and Figure 10. It has been observed that by growing the values of both parameters, temperature curves of nanofluid and hybrid nanofluid declined. Moreover, temperature curves are enhanced while boosting the values of Nb,M,Nt and Bi as shown in Figure 11, Figure 12, Figure 13 and Figure 14. This type of behaviour is explained by the fact that enhancing the Brownian motion parameter causes an increase in the random motion of fluid particles. This increase in random motion raises the mean kinetic energy of fluid particles, which raises the temperature of the simple nanofluid and hybrid nanofluid. Physical, thermal Biot number proves that an increase in the energy gradient toward the surface results in a reduction in the thickness of the thermal boundary layer.

**Figure 15:**
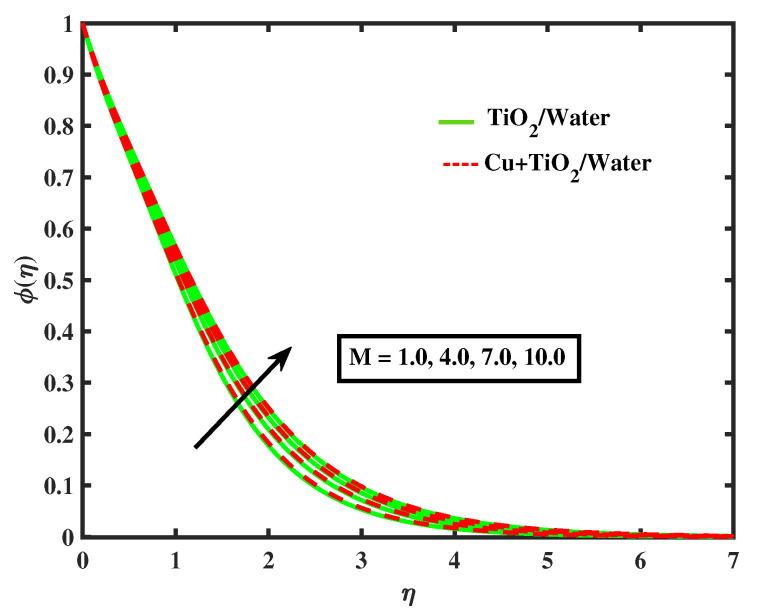
Variations of ϕ versus *M*.

**Figure 16:**
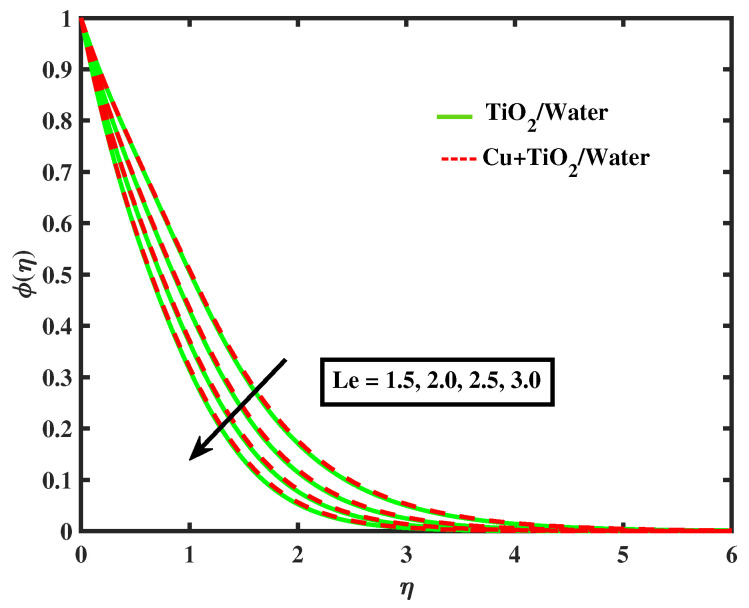
Variations of ϕ vesus Le.

**Figure 17:**
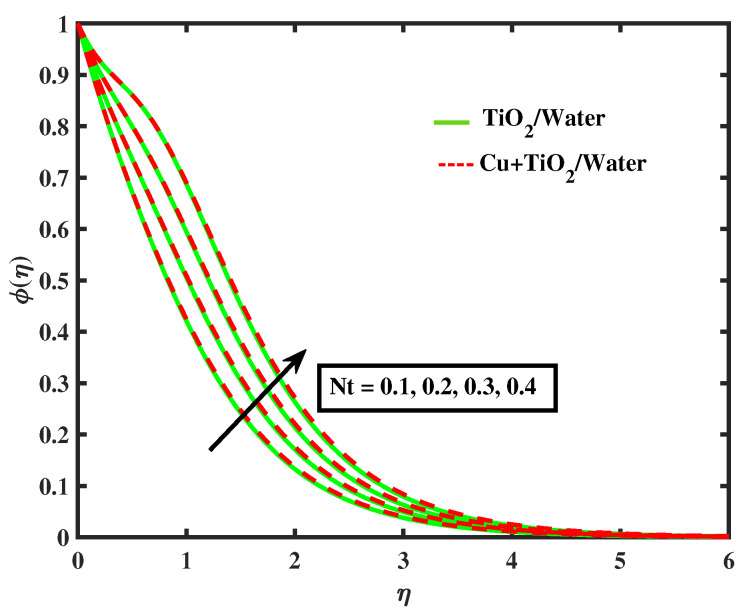
Variations of ϕ versus Nt.

**Figure 18:**
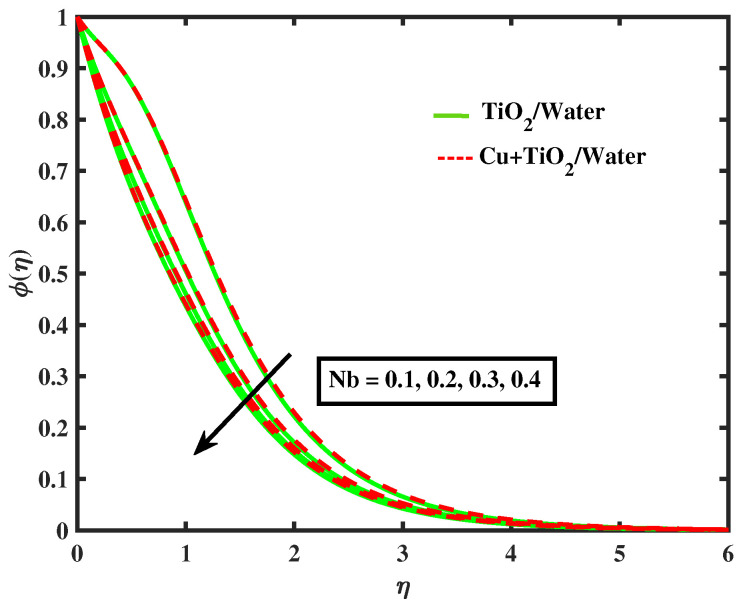
Variations of ϕ versus Nb.

**Figure 19:**
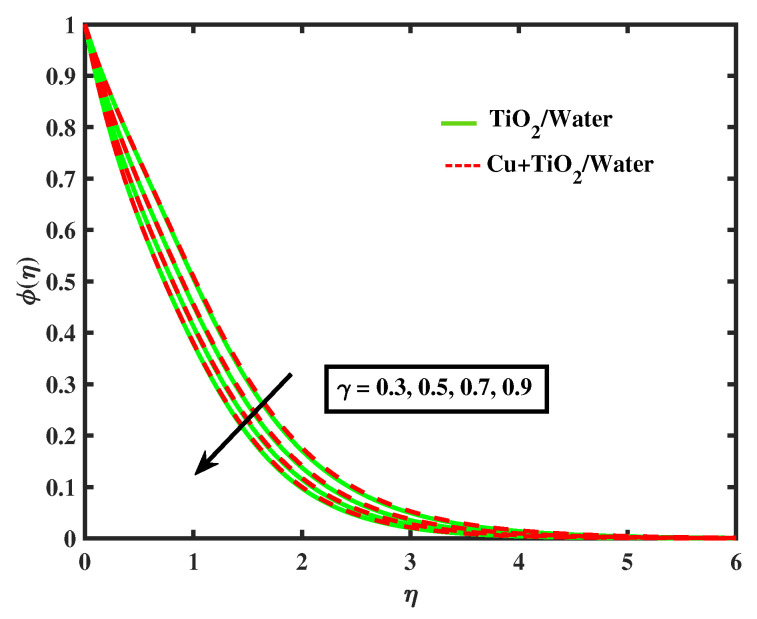
Variations of ϕ versus γ.

Figure 15 and Figure 16 display the impacts of magnetic parameter and Lewis number on concentration profiles of $TiO_{2}$/Water and $Cu+TiO_{2}$/Water. From these figures it has been visualized that concentration of both fluids enhanced with the increment in *M* while opposite behavior is observed for higher values of Le. Figure 17, Figure 18 and Figure 19 demonstrate the concentration of simple nanofluid and hybrid nanofluid flow for parameters Nt,Nb, and γ, respectively. Figure 17 shows increasing behavior of $TiO_{2}$/Water and $Cu+TiO_{2}$/Water concentration profiles for boosting values of Nt whereas decreasing behavior has been observed for growing values of Nb and γ as shown in Figure 18 and Figure 19. In reality, when Nt increases, the fluid particles accelerate rapidly, resulting in an increase in kinetic energy that causes the boundary layer to grow. Physically, the random acceleration decreases as the quantity of Nb grows, the flow of fluid particles from peak areas to bottom regions improves fast.

**Figure 20:**
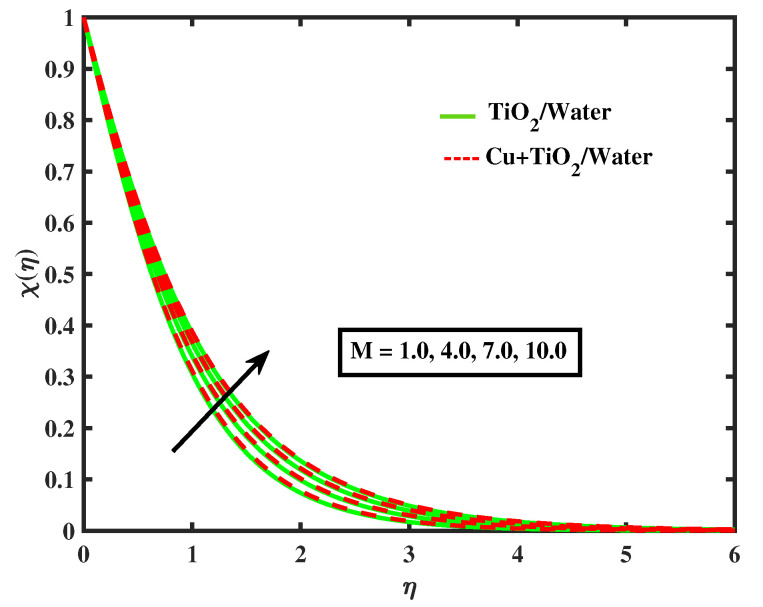
Variations of χ versus *M*.

**Figure 21:**
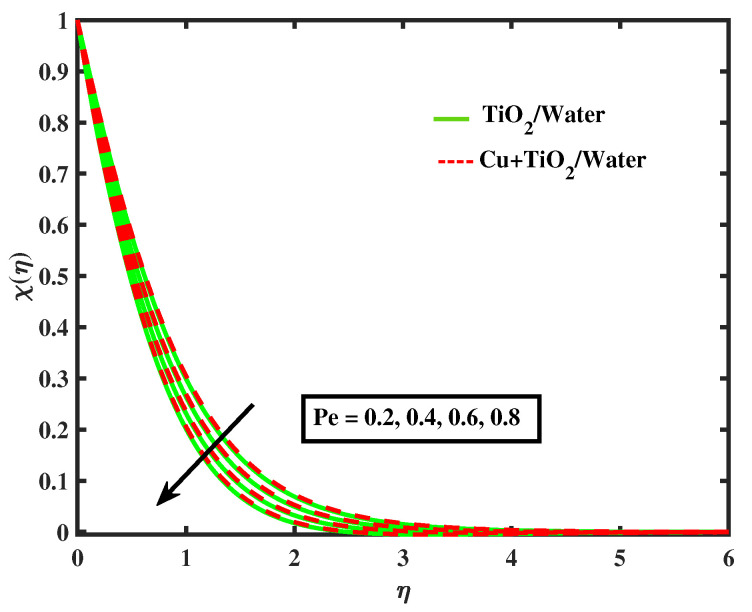
Variations of χ versus Pe.

**Figure 22:**
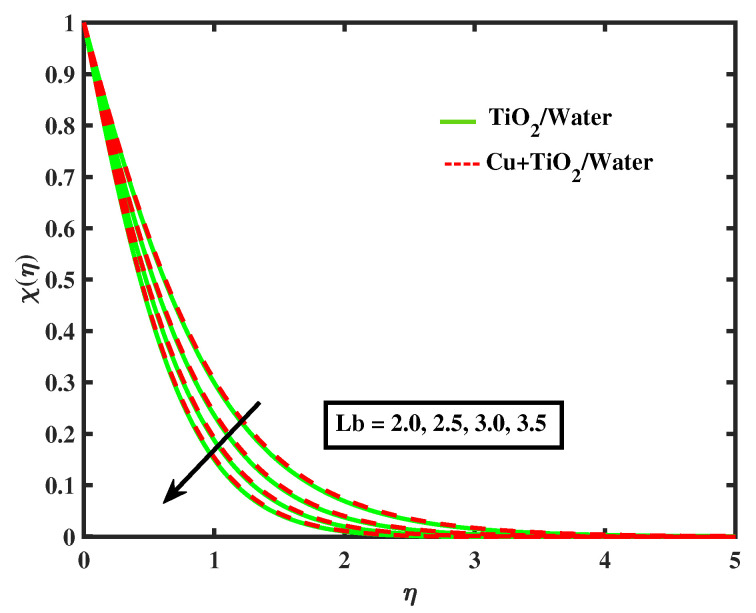
Variations of χ versus Lb.

**Figure 23:**
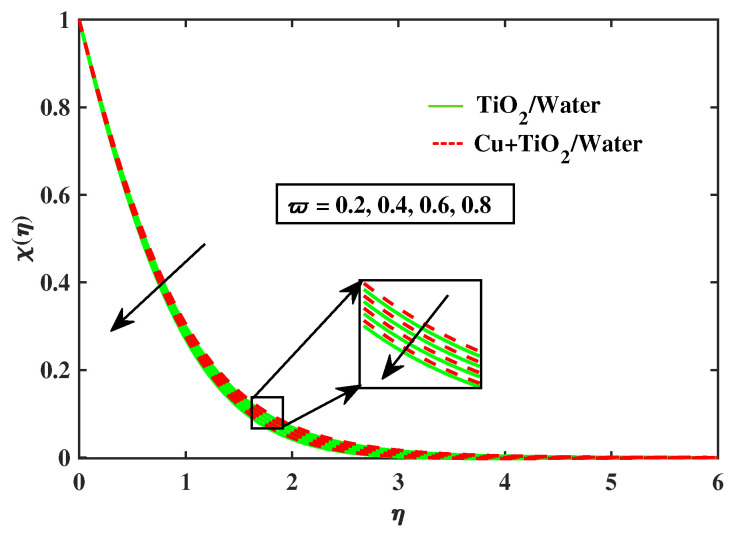
Variations of χ versus ϖ.

Figure 20, Figure 21, Figure 22 and Figure 23 portray behavior of motile microbe profiles (χ(η)) by varying parameters for both fluids. The impacts of the bio-convected Peclet number (Pe) and the magnetic parameter (*M*) has been illustrated in Figure 20 and Figure 21. It is observed that the microorganisms boosts along with *M*, but it declines with the increase in Pe. Figure 22 and Figure 23 depict the profiles of microbes for Lb and ϖ. It is noted that the profiles of microbes decrease for the boosted values of Lb and ϖ. Table 5 signifies the numerical values of Nusselt number and local skin friction coefficient versus different values of parameters.

## 5. Conclusions

The hybrid nanofluid flow has been studied with bioconvection and chemical reaction over an expanding surface. This paper solves the PDEs system through similarity transformation and obtains ordinary differential equations. The numerical method RKF-45 package built-in MATLAB is applied to solve these ODEs. The significant findings are summarized as follows:

- The velocity profile of both fluids has positive behavior for large values of Prandtl fluid parameters and opposite behavior for the angle of inclination, magnetic parameter, and bioconvection Rayleigh number.
- The boosting valuation of Prandtl fluid parameters, simple nanofluid, and hybrid nanofluid temperature profiles are declined.
- Temperature profiles are enhanced for the large values of the magnetic parameter, thermophoresis parameter, Brownian motion parameter, and Biot number. Also, the temperature profiles of hybrid nanofluids are more significant than those of simple nanofluids.
- The behavior of concentration of simple nanofluid and hybrid nanofluid is negative for the increasing values of Brownian motion, chemical reaction, and Lewis parameters.
- The volumetric concentration of simple and hybrid nanofluids has positive nature for a higher valuation of magnetic and thermophoresis parameters.
- The negative feature of microorganisms of simple and hybrid nanofluids is observed for Peclet and bioconvection numbers.
- Hybrid nanofluid results are more prominent than the nanofluid flow.
- The skin friction coefficient of both fluids has a decreasing trend for boosted inputs of $\alpha_{2},\alpha ,Nt$ while an increasing trend for $\alpha_{1},Nb,Bi$.
- The Nusselt number rises for $\alpha_{1},\alpha_{2},Bi$, and the decreasing trend is observed for M,α,Nt,Nb.

## Figures and Tables

**Table 1:** Thermo-physical attributes of hybrid nanofluid.

Properties	Nanofluid	Hybrid Nanofluid
Viscosity	$\mu_{nf}=\frac{\mu_{f}}{{\left(1-\phi \right)}^{2.5}}$	$\mu_{hnf}=\frac{\mu_{f}}{{\left(1-\phi_{1}\right)}^{2.5}{\left(1-\phi_{2}\right)}^{2.5}}$
Density	$\rho_{nf}=\left(\phi \left(\frac{\rho_{s}}{\rho_{f}}\right)+\left(1-\phi \right)\right)\rho_{f}$	$\rho_{hnf}=\left(1-\phi_{2}\right)\left(\phi_{1}\left(\frac{\rho_{s1}}{\rho_{f}}\right)+\left(1-\phi_{1}\right)\right)\rho_{f}$
		$+\phi_{2}\rho_{s2}$
Heat Capacity	${\left(\rho c_{p}\right)}_{nf}=\left(\left(1-\phi \right)+\phi \left(\frac{{\left(\rho c_{p}\right)}_{s}}{{\left(\rho c_{p}\right)}_{f}}\right)\right){\left(\rho c_{p}\right)}_{f}$	${\left(\rho c_{p}\right)}_{hnf}={\left(\rho c_{p}\right)}_{f}\left(1-\phi_{2}\right)\left(\left(1-\phi_{1}\right)+\phi_{1}\left(\frac{{\left(\rho c_{p}\right)}_{s1}}{{\left(\rho c_{p}\right)}_{f}}\right)\right)$
		$+\phi_{2}{\left(\rho c_{p}\right)}_{s2}$
Thermal conductivity	$\frac{k_{nf}}{k_{f}}=\frac{-\left(m-1\right)\phi \left(k_{f}-k_{s}\right)+k_{s}+\left(m-1\right)k_{f}}{\phi \left(k_{f}-k_{s}\right)+k_{s}+\left(m-1\right)k_{f}}$	$\frac{k_{hnf}}{k_{bf}}=\frac{k_{s2}+\left(m-1\right)k_{bf}-\left(m-1\right)\phi_{2}\left(k_{bf}-k_{s2}\right)}{k_{s2}+\left(m-1\right)k_{bf}+\phi_{2}\left(k_{bf}-k_{s2}\right)}$
		where, $\frac{k_{bf}}{k_{f}}=\frac{k_{s1}+\left(m-1\right)k_{f}-\left(m-1\right)\phi_{1}\left(k_{f}-k_{s1}\right)}{k_{s1}+\left(m-1\right)k_{f}+\phi_{1}\left(k_{f}-k_{s1}\right)}$

**Table 2:** Thermo-physical attributes of two nanoparticles and water.

Nanoparticles/Base Fluid	Cu	TiO${}_{2}$	H${}_{2}$O
ρ (kg · m${}^{-3}$)	8933	4250	997.1
$C_{p}$ (J · kg${}^{-1}$·K${}^{-1}$)	385	686.2	4179
*k* (W · m${}^{-1}$ · K${}^{-1}$)	401	8.9538	0.613
σ ($\Omega^{-1}$ · m${}^{-1}$)	59.6	0.125	5.5

**Table 3:** Comparison of $-f^{\prime \prime }\left(0\right)$ for different values of $M,\alpha_{1}=1,\alpha =90^{0}$ and the remaining parameters are zero.

*M*	Gireesha et al. [34]	Jalil et al. [35]	Ali et al. [36]	Our Outcomes
0.0	1.000	1.000000	1.0000080	1.00000837
0.2	1.095	1.095445	1.0954458	1.09544603
0.5	1.224	1.224745	1.2247446	1.22474492
1.0	1.414	1.414214	1.4142132	1.41421356
1.2	1.483	1.483240	1.4832393	1.48323970
1.5	1.581	1.581139	1.5811384	1.58113883
2.0	1.732	1.732051	1.7320504	1.73205081

**Table 4:** Comparison of $-\theta^{{}^{\prime }}\left(0\right)$ for defferent values of $Pr,\alpha_{1}=1,Bi\to \infty$ and set all other parameters equal to zero.

*Pr*	Wang [37]	Khan and Pop [38]	Srinivasulu and Goud [40]	Our Outcomes
0.7	0.4539	0.4539	0.4539	0.4544473
2.0	0.9114	0.9113	0.9113	0.9113528
7.0	1.8954	1.8954	1.8954	1.8954004
20.0	3.3539	3.3539	3.3539	3.3539018
70.0	6.4622	6.4621	6.4621	6.4621975

**Table 5:** Numerical values of skin friction coefficient and Nusselt number of mono and hybrid nanofluid for different values of parameters.

*M*	$\alpha_{1}$	$\alpha_{2}$	α	*Nb*	*Nt*	*Bi*	Rex0.5C˜fx	Rex0.5C˜fx	Rex−0.5N˜hx	Rex−0.5N˜hx
							Mono Case	Hybrid Case	Mono Case	Hybrid Case
1.0	0.5	0.5	30.0	0.2	0.2	0.5	−1.6989	−1.7846	0.4146	0.4119
1.2							−1.7437	−1.8288	0.4141	0.4115
1.4							−1.7882	−1.8727	0.4137	0.4110
1.0	0.4						−1.7298	−1.8182	0.4124	0.4096
	0.6						−1.6813	−1.7645	0.4164	0.4138
	0.8						−1.6752	−1.7546	0.4192	0.4167
	0.5	0.6					−1.7894	−1.8803	0.4155	0.4129
		0.7					−1.8708	−1.9662	0.4163	0.4137
		0.8					−1.9449	−2.0444	0.4169	0.4144
		0.5	45.0				−1.9200	−2.0027	0.4123	0.4097
			60.0				−2.1341	−2.2145	0.4101	0.4075
			75.0				−2.2872	−2.3660	0.4086	0.4060
			30.0	0.4			−1.6414	−1.7267	0.3275	0.3261
				0.6			−1.5777	−1.6625	0.2337	0.2335
				0.8			−1.5138	−1.5980	0.1494	0.1498
				0.2	0.1		−1.6973	−1.7829	0.4256	0.4228
					0.3		−1.7001	−1.7859	0.4027	0.4002
					0.4		−1.7008	−1.7867	0.3898	0.3876
					0.2	1.0	−1.6568	−1.7420	0.5619	0.5588
						1.5	−1.6342	−1.7190	0.6331	0.6299
						2.0	−1.6203	−1.7049	0.6745	0.6713

## Data Availability

The data used to support the findings of this study are included within the article.

## Nomenclature

The following Nomenclature are used in this manuscript:

*T*	dimensionless temperature
$T_{w}$	fluid temperature at wall
*C*	dimensionless concentration
$C_{w}$	concentration at wall
$T_{\infty }$	temperature far from the sheet
λ	mixed conviction parameter
*N*	density of the motile microorganisms
$N_{w}$	motile microorganisms’ density at wall
$C_{\infty }$	concentration away from the surface
$\alpha_{1}$	Prandtl fluid parameter
$\tilde{C}f_{x}$	skin friction at x-direction
$\left(\tilde{u},\tilde{v}\right)$	components of velocity
$\alpha_{2}$	elastic parameter
${\tilde{u}}_{w}$	velocity of stretching sheet
Nr	buoyancy ratio parameter
ϖ	bio convection constant
α	inclination angle
$D_{m}$	microorganism coefficient
$\nu_{f}$	kinematic viscosity of nanofluid
Lb	bioconvection Lewis number
Nt	parameter of thermophoresis
$K_{r}$	thermal conductance
$D_{T}$	coefficient of thermophoretic diffusion
$D_{B}$	Brownian diffusion coefficient
$w_{c}$	maximum cell swimming speed
γ	chemical reaction parameter
*b*	chemotaxis constant
Pr	Prandtl number
$\tilde{N}u_{x}$	Nusselt number
Nc	bioconvection Rayleigh number
Bi	thermal Biot number
λ	mixed convection parameter
Le	Lewis number
$\rho_{f}$	nanofluid density
η	dimensionless variable
Nb	Brownian motion parameter
*M*	magnetic parameter
$Re_{x}$	Reynold’s number
Pe	Peclet number
$g^{\prime }$	acceleration due to gravity
$B_{0}^{2}$	uniform magnetic field strength
$\nu_{hnf}$	kinematic viscosity of hybrid nanofluid
$\rho_{p}$	nanofluid density
$\rho_{m}$	motile microorganism density
${\bar{\alpha }}_{hnf}$	thermal diffusivity of hybrid nanofluid
$N_{\infty }$	ambient density of the motile microorganisms
τ	ratio of the specific heat capacities
